# Carfilzomib and the cardiorenal system in myeloma: an endothelial effect?

**DOI:** 10.1038/bcj.2015.112

**Published:** 2016-01-15

**Authors:** A Rosenthal, J Luthi, M Belohlavek, K M Kortüm, F Mookadam, A Mayo, R Fonseca, P L Bergsagel, C B Reeder, J R Mikhael, A K Stewart

**Affiliations:** 1 Division of Hematology Oncology, Mayo Clinic Arizona, Scottsdale, AZ, USA

## Abstract

Carfilzomib (Cfz) has been associated with an ~5% incidence of unexplained and unpredictable cardiovascular toxicity in clinical trials. We therefore implemented a detailed, prospective, clinical cardiac and renal evaluation of 62 Cfz-treated myeloma patients, including serial blood pressure (BP), creatinine, troponin, NT-proBNP and pre- and post-treatment echocardiograms, including ejection fraction (EF), average global longitudinal strain and compliance. Pre-treatment elevations in NT-proBNP and BP, as well as abnormal cardiac strain were common. A rise in NT-proBNP occurred frequently post-treatment often without corresponding cardiopulmonary symptoms. A rise in creatinine was common, lessened with hydration and often reversible. All patients had a normal EF pre-treatment. Five patients experienced a significant cardiac event (four decline in EF and one myocardial infarction), of which 2 (3.2%) were considered probably attributable to Cfz. None were rechallenged with Cfz. The ideal strategy for identifying patients at risk for cardiac events, and parameters by which to monitor for early toxicity have not been established; however, it appears baseline echocardiographic testing is not consistently predictive of toxicity. The toxicities observed suggest an endothelial mechanism and further clinical trials are needed to determine whether or not this represents a class effect or is Cfz specific.

## Introduction

Therapies such as proteasome inhibitors and immunomodulatory drugs have improved overall response rates and survival in multiple myeloma (MM) patients over the last decade. Bortezomib is a first-generation reversible proteasome inhibitor active both as a single agent and in combination. Although infrequent, bortezomib has been associated with cardiotoxicity in animal models^1^ and in clinical experience.^2, 3, 4^

Carfilzomib (Cfz) is a next-generation proteasome inhibitor that binds irreversibly, resulting in sustained inhibition of the proteasome with minimal neurotoxicity.^5, 6^ Cfz has shown activity as both a single agent and in combination for patients with relapsed myeloma.^7, 8, 9^ Early-phase I/II studies reported a low but reproducible incidence of cardiorenal toxicity, including hypertension (HTN), pre-renal failure, congestive heart failure and ischemic heart disease.^10^

In a pooled safety analysis from four phase II trials (*n*=526), Cfz was associated with any grade dyspnea (42%), HTN (14%), renal insufficiency (24%), peripheral edema (24%) and cardiac events (22% (7.2% congestive heart failure)).^11^ Cumulatively, 73.6% of patients experiencing these toxicities had a previous cardiovascular event and 70% had baseline risk factors for cardiac disease.^11^ Recently, a phase III study of Cfz (K) in combination with lenalidomide and dexamethasone (KRd) showed promising activity with rates of cardiotoxicity comparable to previous reports.^12^

The clinical impact of proteasome inhibitor associated cardiotoxicity has not yet been satisfactorily determined and the pathophysiology is poorly understood. There is no standard approach to cardiac work-up, predicting toxicity or managing events when they occur. In this report, we therefore detail a prospective clinical cardiorenal evaluation of 62 Cfz-treated MM patients to gain a better understanding of the impact and to elucidate predictive factors of treatment-related cardiorenal events.

## Methods

Sixty-two patients with MM received Cfz between August 2011 and May 2014 at Mayo Clinic Arizona. Cfz dose, number of cycles and concurrent chemotherapy was recorded. Delivery of hydration pre- and post-treatment was documented. Systolic blood pressure (SBP), creatinine, troponin and NT-proBNP were recorded on days 1 and 2 of cycle 1. NT-proBNP was measured on day 8, whereas creatinine and SBP were collected on day 15. Echocardiograms, with left ventricular ejection fraction (LVEF), average global longitudinal strain and E/e′ ratio were performed at baseline, and following four cycles of Cfz. All parameters were measured prospectively; data were collected and analyzed retrospectively with notable cardiorenal events examined for attribution. Institutional Review Board approval for publication of this deidentified clinical data was obtained.

## Results

atient characteristics are presented in Table 1. Sixty-two patients were included for analysis. The median age was 65 years (range 39–78); 60% were male. Twenty patients were untreated and received Cfz as part of a clinical trial (Cfz with dexamethasone, thalidomide and cyclophosphamide).^13^ Forty-two patients had relapsed disease, with all but one previously treated with bortezomib. Relapsed patients were heavily pre-treated with a median of four prior therapies (range 1–10), including anthracyclines in 21% and autologous stem cell transplant in 50%. In the relapsed group, 19 patients received Cfz alone, 10 received Cfz with cyclophosphamide and 10 received Cfz with an immunomodulatory agent (lenalidomide or pomalidomide). One patient received Cfz with pomalidomide and cyclophosphamide, and two patients received thalidomide with cyclophosphamide. Dexamethasone was given concurrently in 95% of patients (4 mg (23/62), 20 mg (6/620) and 40 mg (30/62)).

Patients received Cfz at 20 mg/m^2^ initially. Doses with subsequent cycles ranged from 27–45 mg/m^2^ depending on the regimen given on standard dosing days.

Eighteen percentage of patients had baseline HTN (mean153 mmHg; range (146–178 mmHg)), 90% of which had been prescribed antihypertensive medication. Systolic HTN was noted in 12/41 patients on day 2 (6/41 or 15%, newly >140) and 10/41 on day 15 (3/41 or 7%, newly >140). Changes in the opposite direction were also seen, with 11% of patients having a >15 mmHg SBP decrease on day 2. Only three patients (two with baseline HTN) had medication adjustments for optimal BP management. Although baseline BP was not generally predictive of future cardiovascular or renal event, it is notable that two hypertensive patients were hospitalized with volume overload during cycle 1.

Hydration (250–500 ml) was delivered to 89% of patients pre-treatment and 63% post-treatment. Baseline creatinine was elevated in 33% of patients on day 1 (mean 1.55 mg/dl; (range 1.3–5.6 mg/dl)). On day 2, 8/54 patients (15%) had a rise from baseline in creatinine of ⩾0.3 mg/dl, five of the eight (63%) also had elevated baseline creatinine. Patients receiving <500 ml hydration on day 1 had a significantly higher creatinine on day 2 (*P*=0.01). Eleven percentage of patients with renal insufficiency experienced improvement in renal function on day 2. Of 26 patients with renal impairment on days 1 or 2, 16 (61.5%) had persistently abnormal creatinine on day 8; 10 with a higher creatinine than baseline. Overall, only 3/45 (6%) patients who had normal creatinine at baseline and received pre-treatment hydration had a ⩾grade 1 creatinine rise.

Sixteen patients had baseline troponin measurements, all of which were normal (<0.01 ng/ml) on day 1. Only two patients, with confounding co-morbidities, subsequently presented with elevated troponins. One patient with cardiac amyloidosis (overlapping with MM) had normal baseline troponin but was hospitalized (cycle 1 day 10) for fluid retention, congestive heart failure and acute kidney injury during which a troponin of 0.029 ng/ml was recorded. A second patient with a history of coronary artery disease, and no baseline troponin, had an non-ST elevation myocardial infarction (troponin 0.181 ng/ml) requiring stent placement on cycle 1 day 2.

**Figure 1:**
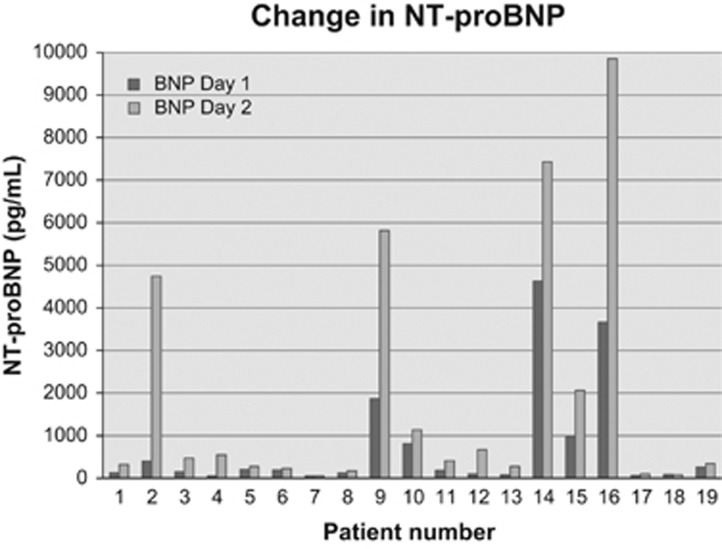
Change in NT-proBNP following treatment with carfilzomib (days 1 and 2).

Twenty-two patients had NT-proBNP measured, with a mean baseline of 565 pg/ml (range <50–3666 pg/ml). After adjusting for age and sex, 82% had abnormal NT-proBNP levels, with concomitant renal insufficiency in 22%. Overall, 16/22 (72%) patients had an increase in NT-proBNP from day 1 to day 2 with an average increase of 1134 pg/ml above baseline (range 36–6187 pg/ml). NT-proBNP continued to rise in 3/16 on day 8. Post Cfz, 52% of patients had a peak NT-proBNP ⩾500 pg/ml and 36% were ⩾1000 pg/ml (mean 551 pg/ml; ([range 82–9853 pg/ml)); 39% with abnormal creatinine. Although only 10 patients had a day 8 NT-proBNP, 4 patients had a continual increase (50% of which had normal or declining creatinine), 3 had declining values and 3 had no previous measurements for comparison. All but one were above normal (range <50- 28 803 pg/ml); (Figure 1).

**Figure 2:**
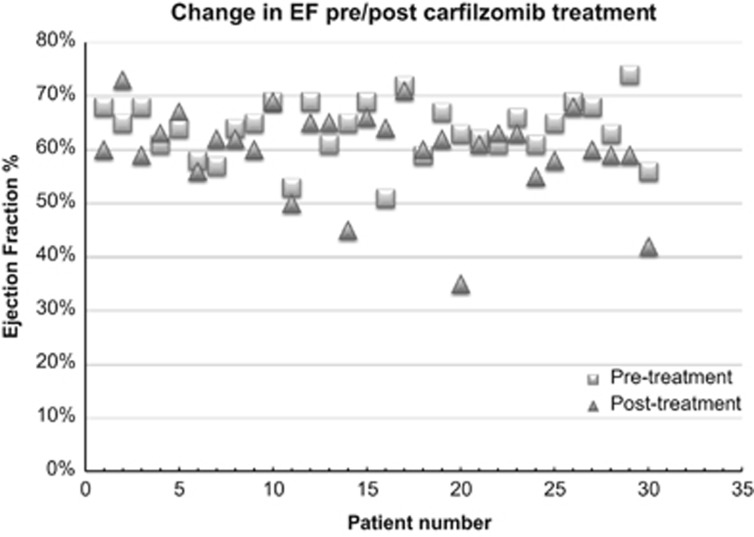
Change in ejection fraction pre-/post-treatment with carfilzomib (*post-treatment refers to ejection fraction assessment following four cycles of treatment with carfizomib).

Echocardiographic measurements, including LVEF, strain and E/e′ ratio, were assessed at baseline and after four cycles of treatment. All 30 patients (19 previously treated (6 with anthracycline exposure); 11 untreated) with baseline echocardiograms had normal LVEF. Three patients (10%) developed impaired systolic function (LVEF <50%); however, two patients had plausible concurrent alternative explanations: one a documented venous thromboembolism/pulmonary embolism and the other had a low EF detected in the setting of multi-lobar pneumonia and systemic sepsis after cycle 2. The third patient had an unanticipated EF decline during cycle 2 detected preoperatively while hospitalized with a pathologic hip fracture. A fourth patient, with a normal EF following cycle 4, was later found to have a diminished EF during cycle 10 while hospitalized with severe sepsis (Figure 2). All four patients that developed impaired systolic function were pre-treated (⩾2 therapies plus stem cell transplant; one prior anthracycline). None continued treatment with Cfz, largely because of progressive disease. In summary, only 1/30 (3%) patients had an unexplained asymptomatic decrease in EF detected in context of concurrent hip fracture, although a declining EF was seen concurrently with other major vascular co-morbidity (pulmonary embolism or acute sepsis in three patients).

Baseline cardiac strain (a measure of left ventricular contractility) was abnormal (⩽−18%) in 14/25 (56%) patients on pre-treatment echocardiogram. As changes in strain can precede a change in EF, post-treatment strain measurements were also obtained. Strain significantly improved in four patients but also worsened in four. Two patients with worsening strain had a corresponding decline in LVEF but remained within the normal range (68/60% 65/58%). Overall, abnormal cardiac strain was very common before therapy, was not significantly changed during therapy and was not predictive for cardiovascular toxicity.

E/e′ ratio was used as a surrogate marker for the left ventricular end diastolic pressure and served as a measure of ventricular compliance. By using a ratio of <15 as normal, 46% of the 28 patients with an E/e′ ratio had abnormal ratios before Cfz treatment. Post-treatment, an equal number of patients, demonstrated worsening or improvement of this parameter.

## Discussion

In this detailed prospective analysis of cardiorenal toxicity following Cfz, significant elevations in SBP and NT-proBNP were common following treatment. Cardiac peptides were frequently elevated at baseline (59%); however, abnormal values are difficult to interpret as they are subject to change based on age, sex, body mass index and renal function.^14, 15^ NT-proBNP rose significantly in 84% of patients immediately following administration of Cfz even in the absence of clinically relevant cardiopulmonary symptoms. Creatinine elevation post-Cfz was common amongst those with baseline renal insufficiency, whereas hydration pre-treatment appeared renal protective in those with normal creatinine at baseline.

Baseline echocardiographic measurements, including LVEF, strain and E/e′ ratio, were not consistently predictive of subsequent cardiotoxicity. Five patients with relapsed/refractory MM had significant cardiac events (Table 2). As described above, four had a decline in LVEF post-treatment, whereas one patient experienced an non-ST elevation myocardial infarction during cycle 1. In only one patient was the decline in EF an isolated event (3%). In all of these patients no further Cfz was administered and follow-up echocardiograms are not available making it difficult to draw any conclusions about recovery of cardiac function, although there is anecdotal data to suggest reversibility.^16^ Our experience correlates with published data of cardiac toxicity and indicates that baseline testing of BNP and cardiac function by echocardiogram is not helpful.^2^ Notably, two of the four cardiac events documented were in patients with amyloid and ischemic heart disease, suggesting that this drug should be used with caution in the presence of baseline cardiac disease and possibly cardiac amyloidosis.

Relapsed/refractory patients are more likely to have co-morbidities. In our experience, significant cardiac events were only observed in previously treated patients. This finding concurs with significantly higher degrees of toxicity in the FOCUS late stage myeloma clinical trial when compared with ASPIRE and ENDEAVOR.^12, 17, 18^

Cardiovascular toxicity, although infrequent, appears to occur early in the course of treatment. Several studies have now demonstrated safety with long-term administration of Cfz.^10, 12, 19^ The long-term tolerability and increasing evidence that cumulative toxicity is uncommon, make Cfz an appealing option for consolidation or maintenance strategies aimed at deepening and prolonging response.^20^

In summary, the cardiorenal impact of Cfz is relatively infrequent yet more common in heavily pre-treated patients and those with baseline cardiorenal dysfunction. The toxicity appears to have an endothelial component—HTN, reversible rise in creatinine (partially preventable by hydration), common acute rise in NT-proBNP and lack of evidence for isolated structural cardiomyopathy. Whether these observations represent a class effect or are unique to Cfz alone is unknown. Prospective controlled studies with longer term follow-up and bortezomib-treated controls are ongoing (ENDEAVOR^18^ (NCT01568866) and CLARION (NCT01818752)).

## Figures and Tables

**Table 1:** Patient Characteristics

*Patient characteristics*	n=*62*
*Age*	
Median	65
Range	39–78

*Sex*	
Male	60%
Female	40%

*Disease status*	
Newly diagnosed	20
Relapsed	42

*# Prior therapies (relapsed patients)*	
Median	4
Range	1–10

Previous treatment	*n*=42
Bortezomib	41 (98%)
Stem cell transplant	31 (50%)
Anthracycline	13 (21%)

**Table 2:** Detailed description of patients with cardiovascular events

*Patient*	*Pre-/post-treatment LVEF*	*Clinical scenario*	*Attributable to Cfz?*
1	65/45%	Multilobar pneumonia	Unlikely
2	61/45% 40% cycle 10	Systemic sepsis	Unlikely
3	63/35%	VTE/PE	No
4	56/42%	Hip fracture (cycle 2)	Probable
5	None/54%	MI/known CAD	Probable

Abbreviations: LVEF, left ventricular ejection fraction; PE, pulmonary embolism; VTE, venous thromboembolism.
